# Epiphytic Terrestrial Algae (*Trebouxia* sp.) as a Biomarker Using the Free-Air-Carbon Dioxide-Enrichment (FACE) System

**DOI:** 10.3390/biology6010019

**Published:** 2017-03-07

**Authors:** Asmida Ismail, Sarah Diyana Marzuki, Nordiana Bakti Mohd Yusof, Faeiza Buyong, Mohd Nizam Mohd Said, Harinder Rai Sigh, Amyrul Rafiq Zulkifli

**Affiliations:** 1 School of Biology, Faculty of Applied Sciences, Universiti Teknologi MARA, Shah Alam 40450, Selangor, Malaysia; sarahdiyana_86@yahoo.com.my (S.D.M.); missdyana92@yahoo.com (N.B.M.Y.); harinder@salam.uitm.edu.my (H.R.S.); amyrulrafiq@gmail.com (A.R.Z.); 2 School of Chemistry and Environment, Faculty of Applied Sciences, Universiti Teknologi MARA, Shah Alam 40450, Selangor, Malaysia; eicha@salam.uitm.edu.my; 3 School of Environmental and Natural Resources, Faculty of Science & Technology, Universiti Kebangsaan Malaysia, Bangi 43600, Selangor, Malaysia; m.n.said@ukm.edu.my

**Keywords:** algae, air pollution, carbon dioxide, FACE, bio-indicator

## Abstract

The increasing concentration of CO_2_ in the atmosphere has caused significant environmental changes, particularly to the lower plants such as terrestrial algae and lichens that alter species composition, and therefore can contribute to changes in community landscape. A study to understand how increased CO_2_ in the atmosphere will affect algal density with minimal adjustment on its natural ecosystem, and the suitability of the algae to be considered as a biomarker, has been conducted. The current work was conducted in the Free-Air-Carbon Dioxide-Enrichment (FACE) system located in Universiti Kebangsaan Malaysia, Bangi, Malaysia. CO_2_ was injected through special valves located along the ring surrounding specimen trees where 10 × 10 cm quadrats were placed. A total of 16 quadrats were randomly placed on the bark of 16 trees located inside the FACE system. This system will allow data collection on the effect of increased CO_2_ without interfering or changing other parameters of the surrounding environment such as the wind speed, wind direction, humidity, and temperature. The initial density *Trebouxia* sp. was pre-determined on 1 March 2015, and the final density was taken slightly over a year later, on 15 March 2016. The exposure period of 380 days shed some light in understanding the effect of CO_2_ on these non-complex, short life cycle lower plants. The results from this research work showed that the density of algae is significantly higher after 380 days exposure to the CO_2_-enriched environment, at 408.5 ± 38.5 × 10^4^ cells/cm^2^, compared to the control site at 176.5 ± 6.9 × 10^4^ cells/cm^2^ (independent *t*-test, *p* < 0.001). The distance between the trees and the injector valves is negatively correlated. Quadrats located in the center of the circular ring recorded lower algal density compared to the ones closer to the CO_2_ injector. Quadrat 16, which was nearing the end of the CO_2_ valve injector, showed an exceptionally high algal density—2-fold higher than the average density at 796 ± 38.5 × 10^4^ cells/cm^2^. In contrast, Quadrat 9, which was located in the center of the ring (lower CO_2_ concentration), recorded only 277 ± 38.5 × 10^4^ cells/cm^2^, which further supports the previous claim. Based on the data obtained, this study provides useful data in understanding the positive effect of CO_2_ on algal density, in a natural environment, and suggests the use of epiphytic terrestrial algae as a biomarker.

## 1. Introduction

Global warming and climate instability, as predicted, are critical and are currently one of the most discussed issues among scientists around the globe. It has been reported that the cause of these problems are directly associated with air pollutants, especially greenhouse gases that are concentrated in the atmosphere. Among pollutants, anthropogenic carbon dioxide (CO_2_) is found to be one of the most important causes of the global warming and climate instability issue [1]. This is supported by a report released by the World Bank [2] stating that carbon dioxide (CO_2_) is responsible for 59% of greenhouse gases. Mass efforts are being made to discover methods to understand the elevated CO_2_ concentration in the atmosphere, and the problem is still unsolved. Therefore, a number of possible solutions have been suggested, and one of them is the use of microalgae. In Moreira’s and Pires’ study [3], microalgae were utilized to absorb CO_2_ from the atmosphere. This is because algae are able to convert CO_2_ released into organic carbon through photosynthesis, which leads to the production of large amounts of biomass [4]. Despite the fact that algae may be used to reduce air pollution, there is paucity of literature on the mid- to long-term effect of CO_2_ on algae—whether they can adapt and survive in the CO_2_-rich environment for a certain period or if the CO_2_ conversion process can only take effect during short-term exposure. This paper will addresses the ability of epiphytic terrestrial algae as mid- to long-term biomarkers. Biomarkers are defined as quantitative measures of changes in the biological system that can be related to exposure to the toxic effects of environmental chemicals [5,6].

The Free-Air-Carbon Dioxide-Enrichment (FACE) system is a method used by ecologists and plant biologists in a specified area to measure the effect of raised CO_2_ concentration in plant growth responses. Researchers believe that measuring the effect of elevated CO_2_ using FACE is a better way to estimate how plant growth will change in the future as the CO_2_ concentration rises in the atmosphere. Another benefit that FACE offers is that it allows the effect of elevated CO_2_ on plants that cannot be grown in small spaces, such as trees, to be measured. Epiphytic terrestrial algae are believed to grow better in habitats with higher CO_2_ concentration. They are known to have the ability to fix the CO_2_ 3–5-fold more than typical trees and crops [7]. In areas with high pollution levels, the growth rate of algae is better, allowing for their colonization on the bark of trees to be highly visible. The aim of this study was to observe the mid-term changes of algal density in a CO_2_-enriched environment using the FACE system.

The objectives of this study are listed as follows:

(1) to study the mid-term exposure of increased CO_2_ on changes in the density of epiphytic terrestrial algae using the FACE system;
(2) to assess the relationship between algal density and the distance from the CO_2_ source.

## 2. Methodology

### 2.1. Site and Environmental Parameter Description

**Figure 1:**
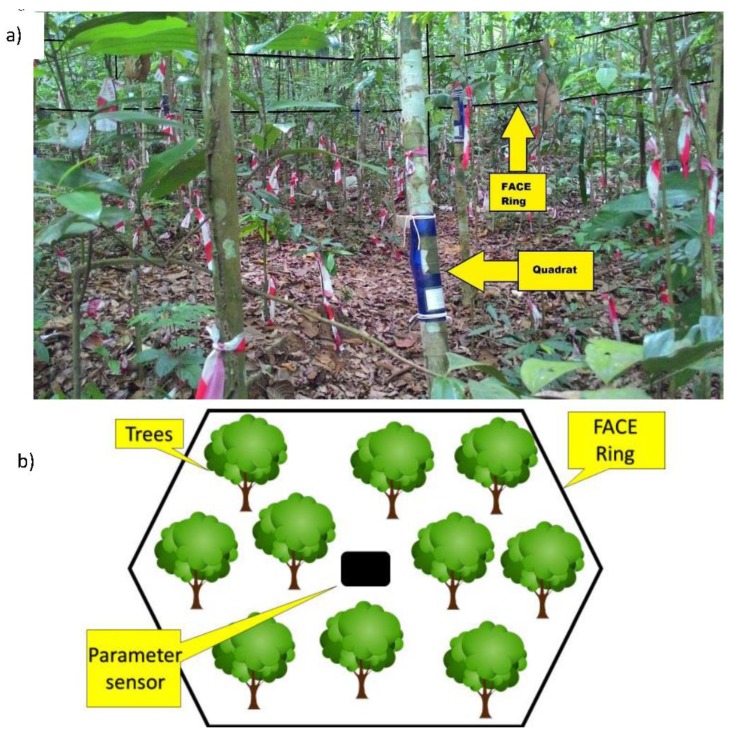
(**a**) Quadrats on trees in the Free-Air-Carbon Dioxide-Enrichment (FACE) system; inner (2 m, 400 ppm), middle (4 m, 440 ppm) and outer (5 m, 630 ppm). (**b**) Overall layout design of the FACE system.

This research work was conducted in the custom-made Free-Air-Carbon Dioxide-Enrichment (FACE) system (Figure 1) located in Universiti Kebangsaan Malaysia (UKM), Selangor, Malaysia. The system covers an area of 55 m^2^, consisting of plants of different species. The quadrats were placed on *Barringtonia racemosa*, a common tree found in Southeast Asia. This system is a unique way to observe the long-term effect of CO_2_ in an open laboratory environment, which also uses the same injection method. The parameters surrounding the environment are in the environments’ natural state, i.e., the parameters are not being changed in any way. The environmental parameters recorded were the wind speed, the wind direction, humidity, and temperature. This system allows other parameters to take place around it naturally while adjusting the level of CO_2_ according to the settings in the FACE system. Thus, this system allows researchers to study the effect of CO_2_ on plants and the living organisms in it without changing their natural conditions.

**Figure 2:**
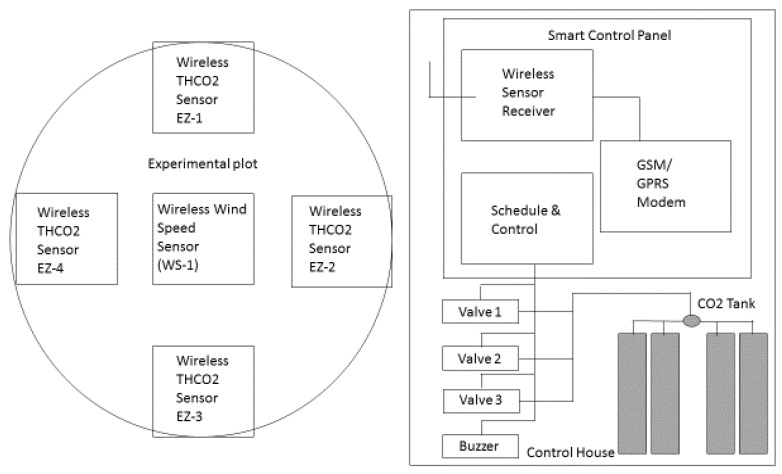
System block of the sensors and working diagram of the FACE.

The features contained in the FACE system are Xbee wireless sensors, a sensor data sender, a CO_2_ scheduler, and a Real Time Clock (RTC) for a date and clock display and a sensor data timestamp. Xbee wireless sensors support four sensor nodes which measure the temperature, humidity, and CO_2_ (EZ sensor nodes) and 1 sensor node which measure the wind speed (WS sensor node). The sensor data sender relays the data to the server immediately after the data is received by SCP. The data will be stored at the Cloud server database and can be viewed remotely from desktops or android devices (Figure 2). The CO_2_ scheduler controls the opening and closing of the valves (where the CO_2_ are being injected) into the area.

In this system, the CO_2_ gas will be injected at one-hour intervals if wind speed is below 15 km/h. There are three valves available in the system. Valve 1 will be in operation for 5 min at the RTC hour, Valve 2 will be in operation for 5 min after 10 min at the RTC hour, and Valve 3 will be active for 5 min after 20 min at the RTC hour. Only one valve will be active at any given time. However, it can also be opened simultaneously using the manual push button. During the injection process, a buzzer will go off to indicate that the process is taking place. The date and time on the LCD can be set up using the menu and keypad.

### 2.2. Systematic Algal Collection and Quantification

**Figure 3:**
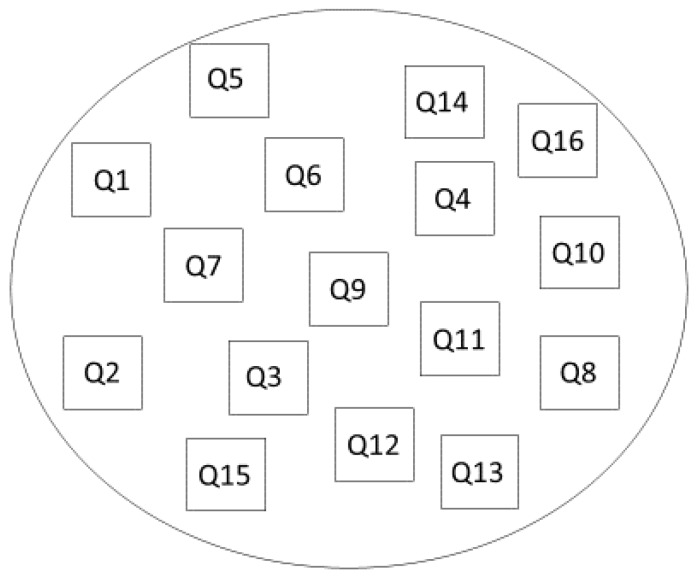
The position of 10 × 10 cm quadrats in the CO_2_ ring injection of the FACE system.

Sixteen trees of the *Barringtonia racemosa* inside the FACE ring were randomly selected, and three quadrats measuring 10 × 10 cm each were placed on the bark of the trees. The experimental layout is illustrated as Figure 3. For the control group, a total of nine quadrats were placed on trees located on the opposite side of the predominant wind, 300 m away from the FACE system. The algal cells inside the quadrats, namely the *Trebouxia* sp., were swabbed to obtain the initial algal density on 1 March 2015. The algal swab contained a mixture of algal species, but since more than 95% are of *Trebouxia* sp., only this species was quantified. The algae in the quadrats were then exposed to the CO_2_ injection through the FACE system for 380 days while quadrats of the control group remained in their natural environment. The density of the algae after the exposure period was collected and quantified on 15 March 2016. The algae were swabbed using wet cotton wool before being stored in 60 mL sterile specimen tubes containing 30 mL of distilled water. The bottles were then stored in the refrigerator at 4 °C. The tube containing algae was shaken vigorously to detach clumpy cells for the quantification process. Ten μL of the sample liquid was then pipetted onto a hemocytometer, ready for algal cell quantification and cell observation under a digital light microscope at 40× magnification. The total of number of cells per mL was calculated using the following formula:

Total number of algal cells in a mL = number of cells counted/10 µL × 1.0 × 10^4^ mL.

## 3. Results and Discussion

### 3.1. Density of Algae after CO_2_ Exposure

**Figure 4:**
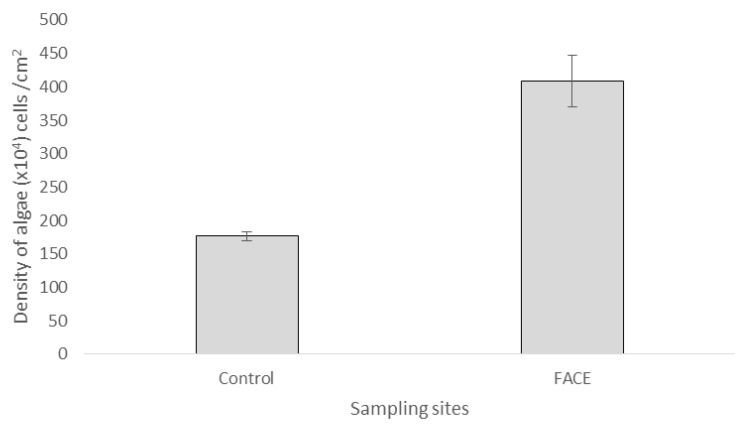
The density of *Trebouxia* sp. recorded from the control and FACE sampling sites after 380 days. Error bars represent standard error of means (SEM).

Figure 4 shows the density of the epiphytic terrestrial algae collected in both control and FACE sampling sites after 380 days of CO_2_ injection. Significantly higher density of epiphytic terrestrial algae was recorded inside the FACE system (408.5 ± 38.5 × 10^4^ cells/cm^2^) as compared to the control site (176.5 ± 6.9 × 10^4^ cells/cm^2^ (independent *t*-test, *p* < 0.001)).

This data implies that the epiphytic terrestrial algae density was affected by the presence of the injected CO_2_ gas molecules. The impact of the CO_2_ molecules on the growth of the epiphytic terrestrial algae is significant as the density of algae in the FACE system was somewhat double that of the control site. CO_2_ is found to be essential for algal photosynthetic activity. Packer [8] stated that algae absorb the extra CO_2_ present in the atmosphere due to CO_2_ injection, capturing it into its biomass and hence increasing in its growth. Besides that, increased external CO_2_ concentration steepens the diffusion gradient for photosynthetic uptake and has been shown to stimulate growth and development in hundreds of photosynthetic organisms [9]. Thus, the abundance CO_2_ gas available in the air allows more gas to be absorbed into the algal cells and used for the photosynthesis process. The mid-term exposure of acidic CO_2_ on the epiphytic terrestrial algae is more beneficial than it is harmful, according to the finding. This indicates that epiphytic terrestrial algae are not negatively affected by the mid-term exposure of acidic CO_2_, thus enabling them as a possible mid- to long-term biomarker. This finding was in agreement with previous studies conducted by other studies [10,11,12].

### 3.2. The Relationship between Algal Density and the Distance of CO_2_ Source

**Figure 5:**
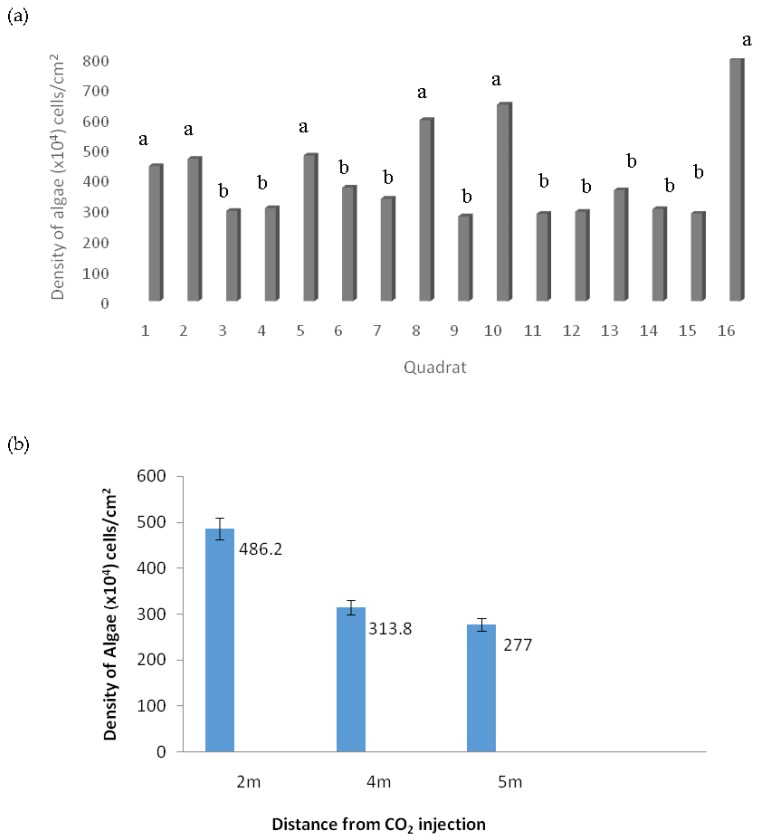
(**a**) Density of epiphytic terrestrial algae of 16 quadrats placed in the ring of the FACE system. (**b**) Mean algal density based on the distance and concentration from CO_2_ injection to quadrats placement. 2 m, inner = 400 ppm; middle, 4 m = 440 ppm; 5 m, outer = 600 ppm. All error bars represent standard error of means (SEM).

Figure 5a shows the density of epiphytic terrestrial algae collected in quadrats located in the FACE system. Based on the figure below, the density of the algae is not uniform throughout the quadrats. Bearing in mind that the FACE system works by taking into account the direction of the prevailing wind, the wind direction and other parameters are thus not being manipulated. Quadrat 16, which is located on the outer area closer to the CO_2_ injection, shows remarkably high algal density (796 ± 38.5 × 10^4^ cells/cm^2^), while the quadrat with the lowest density was Quadrat 9 (farthest from the CO_2_ injection) with only 277 ± 38.5 × 10^4^ cells/cm^2^. Quadrat 1, Quadrat 2, Quadrat 5, Quadrat 8, and Quadrat 10 showed significantly higher algal density compared to other quadrats. This can be best explained by the position of the quadrats in the FACE system (refer to Figure 3). Concurrently, quadrats with significantly higher algal density were located closer to the outer ring of the system; meanwhile, quadrats with lower algal density were positioned farther from the CO_2_ injection ring.

As the CO_2_ gas is injected and dispersed into the air, the quadrats near the ring have better access towards the gas compared to the quadrats placed in the center. In other words, the distance of the algae on the quadrats near the ring to the CO_2_ source is “shorter” compared to the algae on the quadrats in the middle. The short distance between the algae and the CO_2_ source has lower CO_2_ gas dispersal, causing the area to have a higher concentration of CO_2_ compared to the center of the ring where the algae are able to maximize their CO_2_ absorbance. Therefore, the higher CO_2_ absorbance rate increases the rate of photosynthesis of the algae and thus increases their growth. This study was in line with previous studies, which showed that algal density increased as the concentration of CO_2_ increased [13,14]. The findings collected in this research work prove that the distance between the quadrat and the CO_2_ source does play important roles in determining the density of algal cells.

## 4. Conclusions

The conclusion drawn from this study is epiphytic terrestrial algae, particularly of *Trebouxia* sp., can be considered a good mid- to long-term biomarker for rising CO_2_ concentration. This can be seen as CO_2_ considerably triggers higher algal density when the surrounding area contained high CO_2_ concentration. CO_2_, as one of the growth factors for epiphytic terrestrial algae, promotes algal growth, therefore supporting the hypothesis that shorter distances between algae and CO_2_ sources favor the algae.
